# Arthroscopic internal drainage of popliteal cysts using the figure-of four position: a retrospective case series

**DOI:** 10.7717/peerj.20658

**Published:** 2026-01-21

**Authors:** Zhengfeng Mei, Wei Ma, Wentao Lei, Guobiao Pan, Lingzhi Ni

**Affiliations:** Department of Orthopedics, Hangzhou Third People’s Hospital, Hangzhou, China

**Keywords:** Popliteal cysts, Baker’s cysts, Arthroscopy, Internal drainage

## Abstract

**Purpose:**

This study aimed to evaluate the efficacy and safety of arthroscopic internal drainage for popliteal cysts using the figure-of-four position.

**Methods:**

Between January 2016 and June 2024, 61 patients with symptomatic popliteal cysts underwent arthroscopic internal drainage with the knee positioned in a figure-of-four, while intra-articular lesions were managed with synovial debridement, meniscal repair/formation, and removal of loose bodies. Preoperative magnetic resonance imaging confirmed the presence of popliteal cysts and concomitant intra-articular lesions. Operation time and intraoperative blood loss were recorded. Patient satisfaction was evaluated by Rauschning-Lindgren (R-L) grade, Lysholm scale, visual analogue scale (VAS) score preoperatively and postoperatively, and popliteal cyst recurrence.

**Results:**

All patients underwent successful surgery without major vascular and nerve injury. The average operation time was 66.1 ± 9.0 min. The average intraoperative blood loss was 8.34 ± 1.82 ml. All patients were followed up for an average of 13.46 months (5–24 months). No knee joint infection, lower extremity thrombosis, or neurovascular bundle injury were found during follow-up. The VAS score decreased from 5.54 ± 1.19 points preoperatively to 2.75 ± 0.77 points at 1 month postoperatively (*P* < 0.05). The Lysholm knee score improved from 62.71 ± 4.22 points preoperatively to 89.70 ± 2.66 points at 6 months postoperatively (*P* < 0.05). The R-L grade of popliteal cyst symptoms showed significant improvement at 6 months postoperatively compared to the preoperative assessment (*P* < 0.05). Postoperative recurrence of popliteal cysts occurred in five cases without any symptoms (R-L grade 0), and the cyst volume did not increase significantly during follow-up.

**Conclusion:**

Arthroscopic internal drainage of popliteal cysts using the figure-of-four position is a technically feasible and safe procedure that resulted in significant clinical improvement and a low rate of asymptomatic recurrence at short-to-mid-term follow-up. The figure-of-four position appears to be a useful technical adjunct for improving access and working space in the posteromedial compartment, and warrants further investigation.

## Introduction

A popliteal cyst, also known as a Baker’s cyst, is a benign, fluid-filled swelling located in the popliteal fossa. This condition is most prevalent in middle-aged and older populations (Abate et al., 2021). The pathogenesis is strongly associated with underlying intra-articular knee disorders that lead to increased synovial fluid production, including degenerative arthritis, meniscal tears, joint instability, and loose bodies (Kim et al., 2023; Su et al., 2020; Zhang et al., 2021). Clinically, popliteal cysts often present with posterior knee pain, localized swelling, and a palpable mass in the popliteal space. Larger popliteal cysts may compress the popliteal vessels and nerves, potentially leading to ischemia, thrombosis, or peripheral neuropathy (Wang et al., 2019; Sanchez, Conkling & Labropoulos, 2011; Kano & Harada, 2021; de Ruiter et al., 2005). Surgical intervention is recommended for symptomatic popliteal cysts that are refractory to conservative management (Herman & Marzo, 2014). Traditional open surgery requires a large, inverted L- or S-shaped incision, which is associated with significant tissue trauma, prolonged recovery, high risk of cyst recurrence, and potential damage to important nerves and blood vessels (Fritschy et al., 2006; Sansone & De Ponti, 1999; Ma et al., 2023). It has been reported that the main cause of popliteal cyst formation is the “one-way valve” flow mechanism (Nha et al., 2023), where synovial fluid can enter the cyst cavity but cannot return to the joint cavity. Arthroscopic management directly addresses this pathophysiology by allowing surgeons to treat the associated intra-articular pathologies and eliminate the valvular mechanism by widening the cyst’s communication portal (the cyst neck), thereby reducing the risk of recurrence (Nha et al., 2023; Malinowski et al., 2023; Rauschning & Lindgren, 1979; Lysholm & Gillquist, 1982; Singh et al., 2024). Although the arthroscopic approach is well-documented and has demonstrated advantages over open surgery (Sansone & De Ponti, 1999; Nha et al., 2023; Malinowski et al., 2023; Yang et al., 2023), safe and efficient access to the posteromedial compartment remains a significant technical challenge. Techniques that can improve this access are therefore valuable. Previous studies have compared internal drainage to cyst wall resection (Su et al., 2020; Ma et al., 2023; You et al., 2022), but the specific utility of intraoperative positioning to facilitate the procedure has not been the primary focus of clinical reports. This case series aims to detail the application and outcomes of the figure-of-four knee position as a technical maneuver to increase working space in the posteromedial compartment, enhance safety by moving neurovascular structures, and improve the efficiency of arthroscopic internal drainage for popliteal cysts. We performed arthroscopic internal drainage of popliteal cysts in 61 patients from January 2016 to June 2024 using the figure-of-four position. The report is as follows.

## Methods

### Patient selection

**Figure 1:**
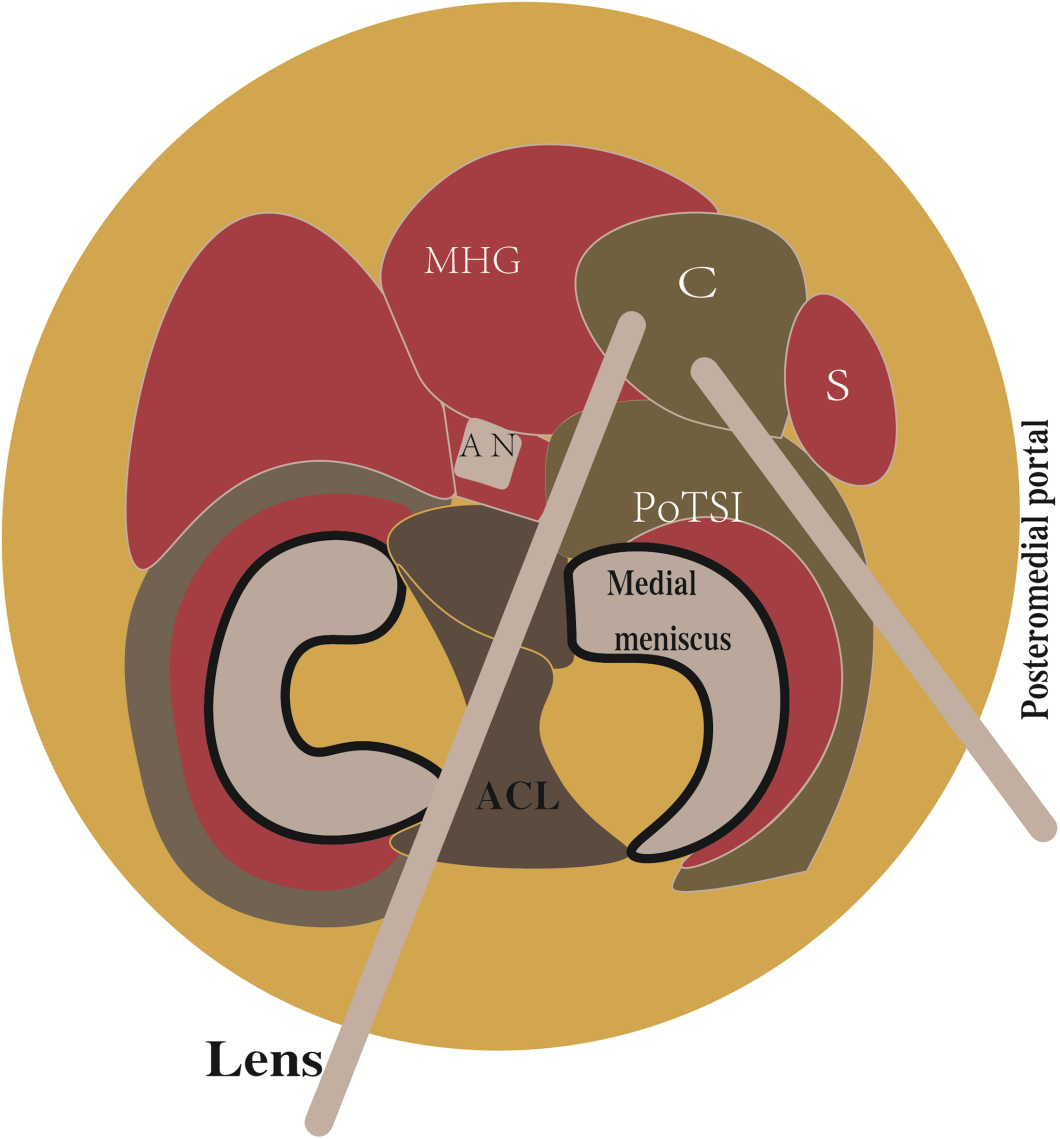
Schematic of the arthroscopic approach for popliteal cyst. The cyst (C) was located between the medial head of the gastrocnemius (MHG) and the semimembranous (S). The arthroscopic lens from the anterolateral portal entered the posteromedial compartment through the gap between the anterior cruciate ligament (ACL) and the medial condyle. The instrument entered the posteromedial compartment through the posteromedial portal.

Because not all popliteal cysts are suitable for arthroscopic treatment, careful preoperative evaluation with magnetic resonance imaging (MRI) is necessary. Axial MRI views are particularly critical for assessing suitability. Popliteal cysts located between the medial head of the gastrocnemius (MHG) and the semimembranosus are suitable for arthroscopic treatment (Fig. 1). The MHG lies between the cysts and neurovascular structures, thus protecting these vital structures from iatrogenic injury. In addition, the arthroscopic lens cannot enter the posteromedial compartment through the severely narrow femoral intercondylar fossa, making the arthroscopic technique infeasible, so special attention should also be paid to the size of the femoral intercondylar fossa in preoperative MRI. Based on our imaging criteria, we established the following inclusion (Table 1) and exclusion criteria for this study. The exclusion criteria were as follows: (1) a history of previous ipsilateral knee or popliteal cyst surgery; (2) significant knee ligamentous instability; (3) specific inflammatory or crystalline arthropathies (*e.g*., rheumatoid arthritis, gouty arthritis) or septic arthritis; (4) pre-existing neuromuscular disorders, psychiatric conditions, or coagulopathies; and (5) incomplete clinical or follow-up data.

**Table 1:** The conditions for arthroscopic Internal drainage of popliteal cysts.

a. Patients aged 15–80 years old. Clinical symptoms: knee pain, swelling, limited knee movement, popliteal swelling, popliteal mass, *etc*. At least 3 months of conservative treatment is unsatisfactory or ineffective.
b. Popliteal cysts with the opening between the medial head of gastrocnemius and the semimembranosus.
c. Rauschning-Lindgren Grades I to III (Rauschning & Lindgren, 1979).
d. No obvious knee deformity, No severely narrow or massively hyperostotic femoral intercondylar fossa.
e. Patients agreed with arthroscopic surgery.

### Patient data

Ethical approval was obtained from Hangzhou Third People’s Hospital (approval number: 2024KA162). The written informed consent were obtained from all the participants. The study was designed in compliance with the Helsinki Declaration.

This was a retrospective study of consecutive patients suffering from symptomatic popliteal cysts. From January 2016 to June 2024, 61 patients (mean age: 61.51 ± 6.93 years, range: 37–73) with popliteal cysts who met the inclusion criteria underwent arthroscopic internal drainage treatment with the knee in figure-of-four position. All patients presented with preoperative symptoms, including knee pain, swelling, limitations in range of motion, and a palpable mass or sensation of fullness in the popliteal fossa. Each patient had undergone a minimum of 3 months of conservative management—including oral non-steroidal anti-inflammatory drugs (NSAIDs), physical therapy, and/or acupuncture—without satisfactory symptom relief. MRI, X-ray examination, and lower limb vascular ultrasound examination were performed before surgery. Axial MRI showed that the popliteal cyst opening was located between the MHG and semimembranosus. Cysts were classified according to Rauschening-Lindgren (R-L) scale criteria. General data of all patients are shown in Table 2.

**Table 2:** Patients demographics, characteristics and intraoperative data.

Age (years)	61.51 ± 6.93
Male\Female (*n*)	21\40
Left\right cysts (*n*)	23\38
Intra-articular pathologies (*n*)	
Meniscus tear (*n*)	49 (80.32%)
Cartilage damage (*n*)	21 (34.42%)
Loose body (*n*)	7 (11.48%)
Knee joint instability (*n*)	4 (6.56%)
Multiple knee pathologies (*n*)	14 (22.95%)
Intra-articular surgery (*n*)	
Partial meniscectomy (*n*)	42 (68.85%)
Meniscal repair (*n*)	7 (11.48%)
Chondroplasty/Microfracture (*n*)	21 (34.42%)
Loose body removal (*n*)	7 (11.48%)
Synovectomy (*n*)	15 (24.59%)
Duration (months)	7.80 ± 6.08
Operation time (min)	66.1 ± 9.0
Intraoperative blood loss (ml)	8.34 ± 1.82
Follow-up period (months)	13.46 + 4.60
Cysts recurrence (*n*)	5
Comorbidities (*n*)	
Osteoarthritis (*n*)	38 (62.22%)
Hypertension (*n*)	19 (31.14%)
Diabetes (*n*)	8 (13.11%)
None (n)	16 (26.23%)

### Surgical technique

**Figure 2:**
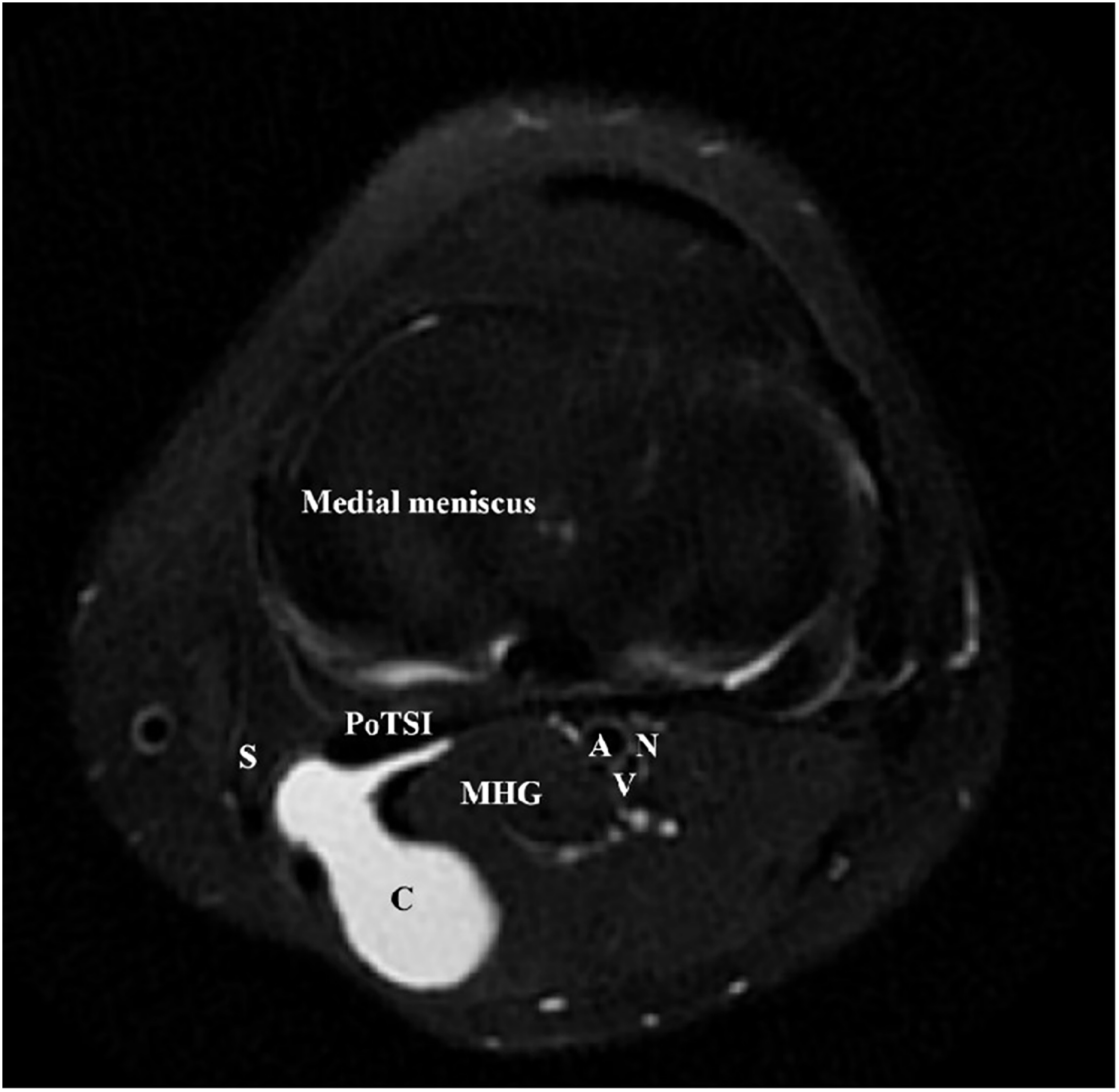
Axial MRI of a left knee demonstrating a popliteal cyst. Popliteal cyst (C) lied in between the gastrocnemius (MHG) and semimembranosus (S). The neurovascular bundle was protected by the medial head of gastrocnemius. The medial head of the gastrocnemius and semimembranosus were labeled for anatomical landmarks.

**Figure 3:**
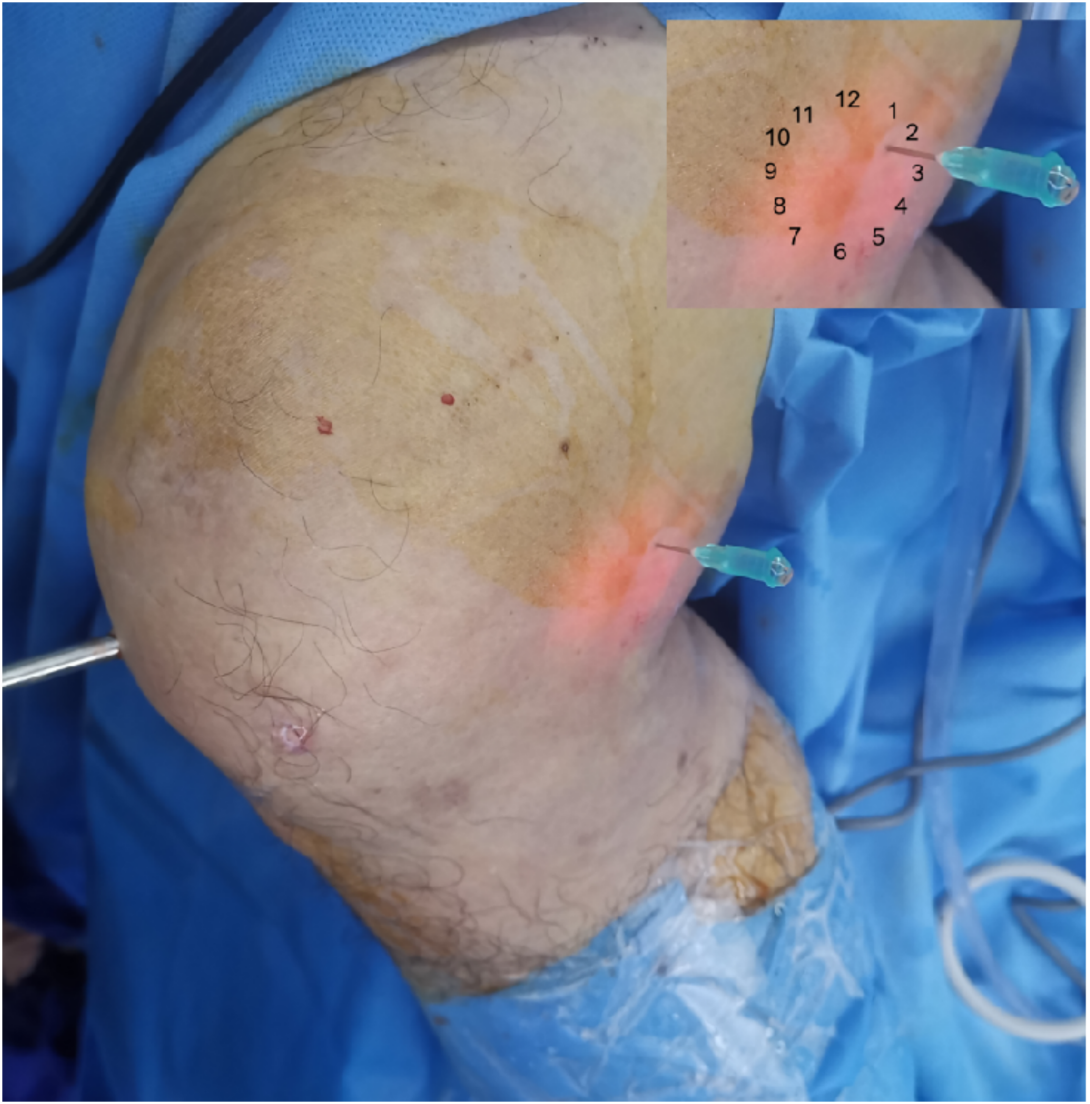
Intraoperative photograph demonstrating the figure-of-four position and portal establishment (right knee). The arthroscopic lens entered the posteromedial compartment. The ankle of the operative leg was placed on the knee of the nonoperative leg, and the operative knee was in a figure-of-four position. Guided by the light from the lens, the needle was inserted at the 2–3 o’clock position (upper right schematic). Successful entry into the posteromedial portal was confirmed by efflux of irrigation fluid through the spinal needle.

**Figure 4:**
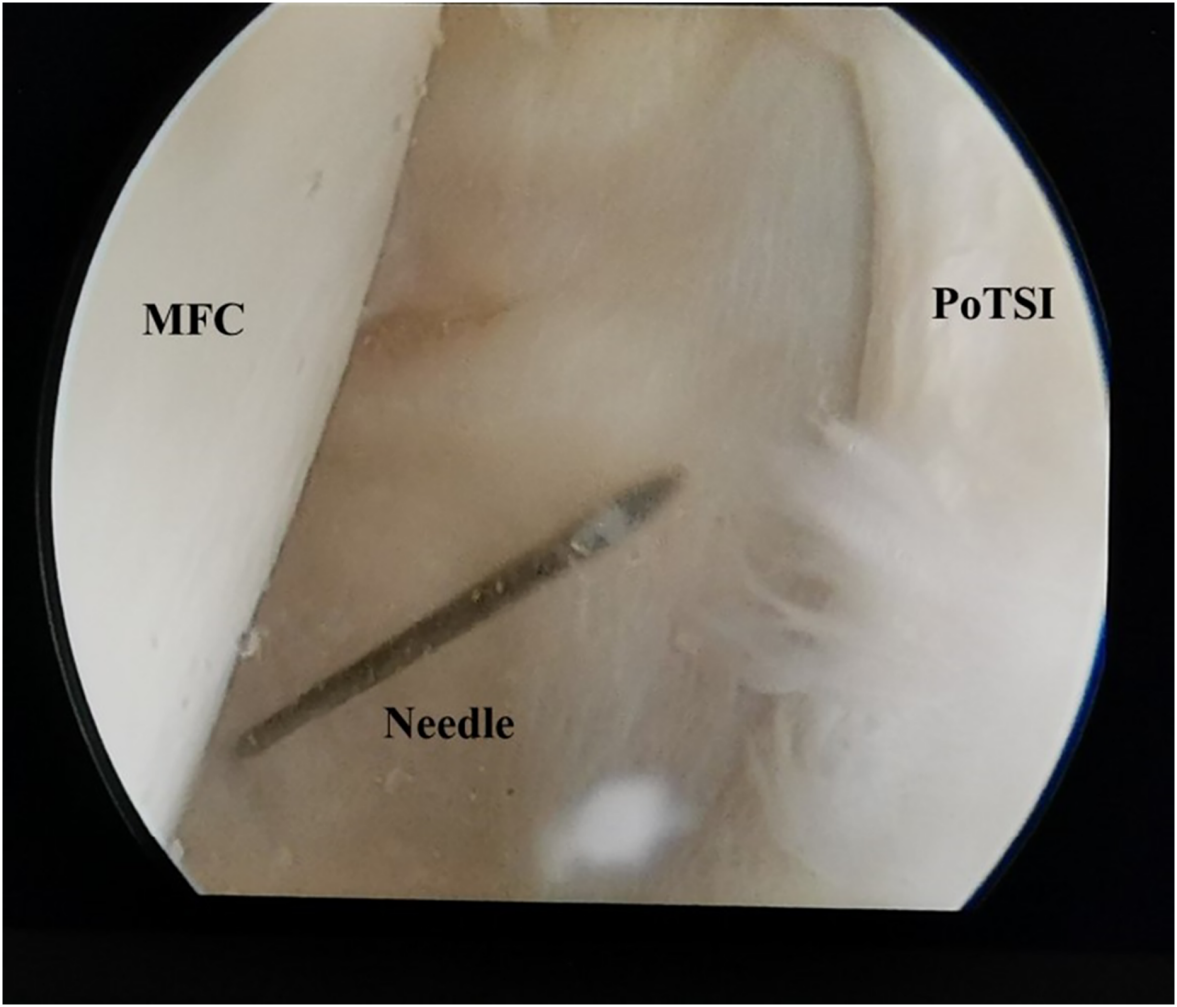
Arthroscopic view of the posteromedial compartment landmarks. With the knee in the figure-of-four position, the arthroscopic view from the posteromedial compartment shows the medial femoral condyle (MFC) and the posterior transverse synovial fold (PoTSI). The spinal needle, introduced under transillumination guidance, is directed toward the PoTSI to confirm the optimal trajectory for portal instrumentation.

**Figure 5:**
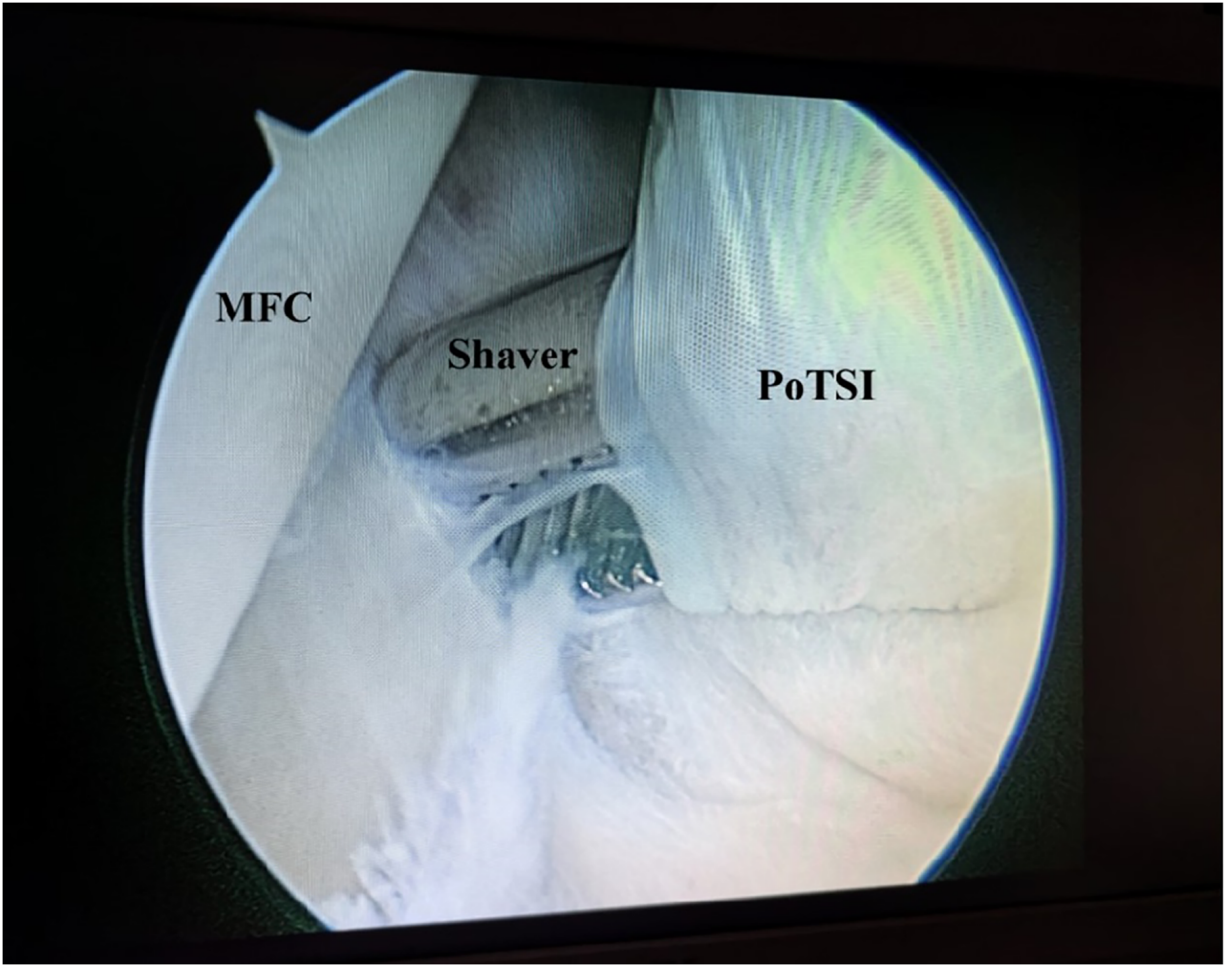
Arthroscopic resection of the posterior transverse synovial fold (PoTSI). Radiofrequency ablation, biting baskets, and shavers were utilized to remove the PoTSI in the posteromedial compartment. Particular attention was paid to preserving the medial meniscus root.

**Figure 6:**
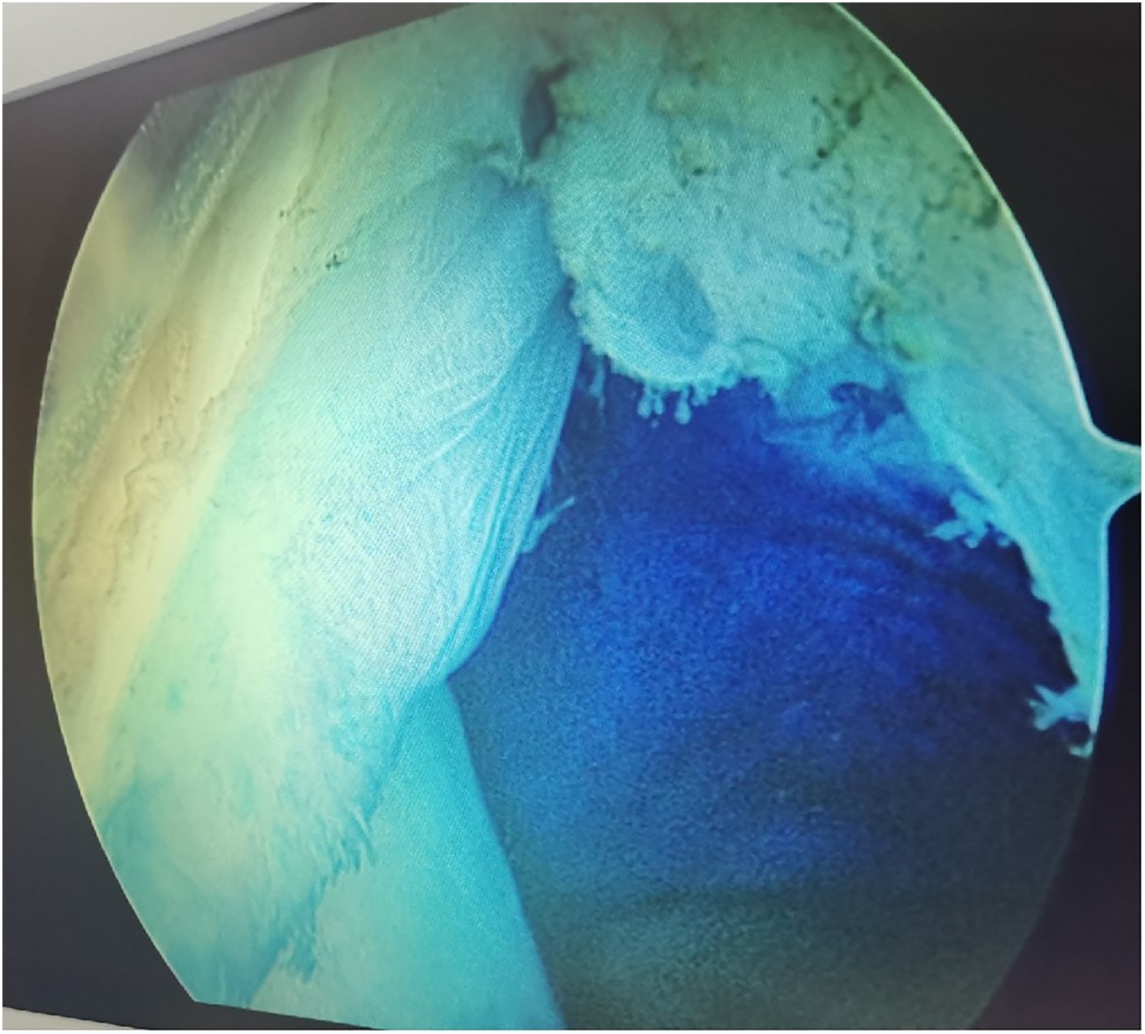
Arthroscopic identification and enlargement of the cyst communication portal. Resection of the PoTSI reveals the inflow of methylene blue dye, confirming the location of the cyst’s communication portal. The medial head of the gastrocnemius (MHG) is exposed. The portal is subsequently enlarged to 1–2 cm to ensure unimpeded flow of synovial fluid and minimize recurrence risk.

**Figure 7:**
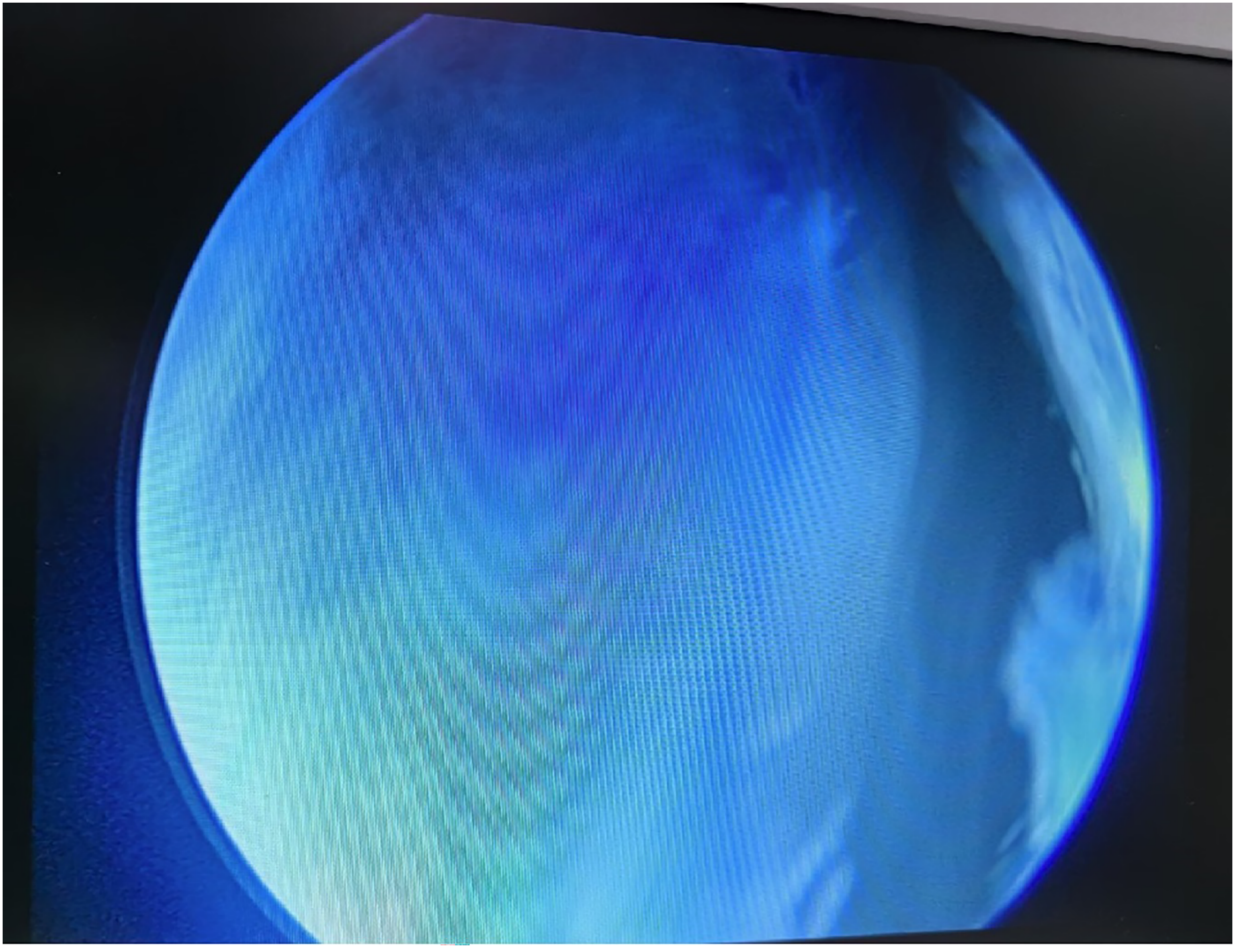
Arthroscopic view of cyst interior. A large amount of blue fluid could be seen. It indicated that synovial fluid can flow freely in the cyst and joint cavity.

All surgical procedures were performed by a single, consistent team of senior orthopedic surgeons, each possessing over 10 years of specialized experience in knee arthroscopy. Patients were positioned supine under combined spinal-epidural or general anesthesia. A pneumatic tourniquet was applied to the proximal thigh of the operative limb (pressure: 60 kPa; maximum duration: 90 min). Standard arthroscopic instrumentation was utilized, including a 4.0 mm 30° arthroscope (Smith & Nephew). Following standard surgical preparation and draping, diagnostic arthroscopy was initiated *via* standard anterolateral and anteromedial portals. All concomitant intra-articular pathologies—such as meniscal tears, synovitis, loose bodies, and chondral lesions—were addressed at this stage. The arthroscopic lens from anterolateral portal entered the posteromedial compartment through the gap between the anterior cruciate ligament (ACL) and medial condyle (Fig. 1). Axial MRI of all patients showed that the popliteal cyst openings were located between the MHG and semimembranosus (Fig. 2). To provide a vivid color marker during the operation and to help precisely locate the cyst opening during arthroscopy, 0.5 ml of methylthioninium chloride was injected into the cysts from the popliteal fossa. The operative knee was then placed into the figure-of-four position by resting the ankle of the operative limb on the contralateral knee (Fig. 3). The posteromedial portal was established by light guidance through the arthroscopic lens (detailed methods in the discussion section). The portal was typically positioned between the 2 and 3 o’clock positions for the right knee (9 and 10 o’clock for the left knee). Correct needle placement was confirmed by the egress of irrigation fluid. The posterior transverse synovial fold (PoTSI) and medial femoral condyle (MFC) were visible, which were important landmarks for localization in the posteromedial compartment (Fig. 4). After scraping the PoTSI using a shaver (in order to avoid damaging the meniscus root), the blue fluid was observed to flow out, which indicated the cyst opening (the opening of the gastrocnemius-semimembranosus bursa) (Fig. 5). After the medial head of the gastrocnemius was exposed, the communication port was further enlarged (1–2 cm) by the shaver, thereby eliminating the “one-way valve” mechanism (Fig. 6). The arthroscopic lens and the shaver entered the cyst cavity along the medial head of the gastrocnemius, requiring resection of all internal septations or diaphragms (Fig. 7). This thorough decompression, including resection of the PoTSI and any internal septations, was essential to minimize the risk of postoperative recurrence. Finally, the instruments were withdrawn, the skin incisions were sutured, and a sterile dressing was applied. Postoperatively, all patients were instructed to wear full-length elastic compression stockings for 2 weeks.

### Postoperative management

Postoperative analgesia was managed for 1 to 2 weeks. The operative limb was wrapped with an elastic compression bandage for 2 weeks. All patients were encouraged to ambulate with crutch support beginning on the first postoperative day. A structured rehabilitation protocol was initiated, emphasizing progressive weight-bearing, quadriceps strengthening, and active and passive range of motion exercises. At 1, 3, and 6 months and 1 and 2 years after operation, the knee function was routinely tested in the outpatient clinic. At each outpatient visit, knee function was assessed clinically. Popliteal ultrasonography or MRI was performed during follow-up if clinical symptoms suggested a potential recurrence.

### Observation content

Preoperative MRI was performed to evaluate intra-articular pathologies and determine the size and location of the cysts. Operation time, intraoperative blood loss, and wound healing time were recorded. Postoperative clinical outcomes were assessed at standardized intervals:

- Pain: measured using the visual analogue scale (VAS) preoperatively and at the 1-month follow-up.
- Knee function: evaluated using the Lysholm knee score preoperatively and at the 6-month follow-up (Sansone & De Ponti, 1999; Lysholm & Gillquist, 1982).
- Cyst symptoms: graded according to the R-L classification system preoperatively and at the 6-month follow-up (Rauschning & Lindgren, 1979).
- Recurrence & complications: cyst recurrence was assessed during follow-up visits *via* popliteal ultrasonography or MRI. All complications—including surgical site infection, hematoma, neurovascular injury, and deep vein thrombosis (DVT)—were meticulously recorded.

### Statistical analysis

All statistical analyses were performed using SPSS software (version 22.0; IBM Corp., Armonk, NY, USA). The normality of quantitative data distribution was assessed using the Shapiro-Wilk test. Data are expressed as mean±standard deviation for normally distributed variables. Preoperative and postoperative VAS and Lysholm scores were compared using paired-sample t-tests. The non-parametric Wilcoxon signed-rank test was used to compare preoperative and postoperative ordinal R-L grades. A *P*-value < 0.05 was considered statistically significant.

## Results

All patients underwent successful surgery without any major neurovascular complications. The mean operation time was 66.1 ± 9.0 min. The mean intraoperative blood loss was 8.34 ± 1.82 ml. Intraoperative findings revealed intra-articular pathologies including meniscal tears (49 cases, 80.32%), cartilage lesions (21 cases, 34.42%), loose bodies (seven cases, 11.48%), instability (four cases, 6.56%), and multiple knee pathologies (14 cases, 22.95%). All patients were followed for a mean duration of 13.46 ± 4.60 months (range: 5–24 months) (Table 2). No postoperative complications—including surgical site infection, DVT, hematoma, or neurovascular injury—were observed during the follow-up period. Five cases of popliteal cyst recurrence were found by B-ultrasound examination, although the cysts were asymptomatic (R-L grade 0) and exhibited no significant volumetric progression on subsequent imaging, thus requiring no further intervention.

The knee VAS score at 1 month postoperatively was significantly lower than the preoperative score (*P* < 0.05). The Lysholm score at 6 months postoperatively was significantly higher than the preoperative assessment (*P* < 0.05). Similarly, the R-L grade for popliteal cyst symptoms also showed significant improvement at 6 months postoperatively compared to preoperative ratings. The detailed preoperative and postoperative data for knee VAS scores, functional scores, and cyst R-L classification are presented in Table 3.

**Table 3:** Preoperative and postoperative VAS and functional scores of knee joint.

	Pre-operation	Post-operation	*P* value
VAS score	5.54 ± 1.19	2.75 ± 0.77	<0.01
Lysholm score	62.71 ± 4.22	89.70 ± 2.66	<0.01
R–L grade (*n*)			<0.01
Grade 0	0	21	
Grade I	15	30	
Grade II	25	10	
Grade III	21	0	

## Discussion

The principal findings of this retrospective case series indicate that arthroscopic internal drainage of popliteal cysts, facilitated by the figure-of-four knee position, is a safe and effective procedure. Short-to-mid-term follow-up data demonstrated that the procedure significantly improved patient pain levels (VAS score), knee function (Lysholm score), and cyst-specific symptoms (R-L grade). The technique demonstrated a low complication rate and an 8.2% (5/61) asymptomatic recurrence rate. Furthermore, we found that the figure-of-four position provides significant technical advantages. It subjectively facilitated superior access to the posteromedial compartment, enhanced procedural safety by increasing the working space and displacing neurovascular structures away from the surgical field, and improved the overall efficiency of the arthroscopic internal drainage procedure.

Arthroscopic management has become a widely accepted treatment for symptomatic popliteal cysts. Sansone & De Ponti (1999), and Ishii et al. (2023) found that the surgical success rate of arthroscopic treatment of popliteal cysts was 95%. Not all popliteal cysts are suitable for arthroscopic treatment. Key determinants of success include the cyst’s anatomical location and the surgeon’s technical proficiency. Popliteal cysts suitable for arthroscopic treatment are usually located between the MHG and the semimembranosus tendon (Wu & Xu, 2022; Goto & Saku, 2020). Since the medial gastrocnemius muscle is in an intermediate position between the neurovascular bundle and cyst, it can be clearly identified intraoperatively to avoid damage to the neurovascular bundle. Similarly, surgical technique is equally crucial. The arthroscopic lens from the anterolateral portal entered the posteromedial compartment through the gap between the ACL and medial condyle to address the popliteal cysts (Fig. 1). The PoTSI and MFC, which were important landmarks of the posteromedial compartment, were visible after the arthroscopic lens entered the posteromedial compartment (Fig. 4). We advocate for the use of the figure-of-four position (achieved by placing the ankle of the operative limb on the contralateral knee) to facilitate this procedure (Fig. 3). This position subjectively increases the working space within the posteromedial compartment and displaces neurovascular structures away from the surgical field, thereby enhancing safety and reducing the risk of inadvertent damage. Furthermore, this rotation externally rotates the tibia and elevates the medial side of the knee, significantly improving ergonomics for the surgeon. Successful establishment of the posteromedial portal is a critical step. We recommend an inside-out technique using transillumination to guide placement. When conceptualizing the illuminated area on the posteromedial skin as a clock face (with the cephalad position at 12 o’clock), the optimal portal location is between the 2 and 3 o’clock positions for the right knee, and between 9 and 10 o’clock for the left knee. A spinal needle should be introduced perpendicular to the skin and directed toward the PoTSI to confirm the optimal trajectory (Fig. 4). Once access is secured, the PoTSI is carefully resected using a shaver or basket forceps, with meticulous care taken to preserve the root of the medial meniscus (Fig. 5). Resection of the PoTSI revealed the inflow of methylene blue dye, confirming the location of the cyst’s communication portal (gastrocnemius semimembranosus cyst opening). Further enlargement of the cyst opening to 1–2 cm ensures the free flow of synovial fluid between the joint and the cyst, a step fundamental to reducing recurrence risk (Figs. 6, 7). In cases of multiloculated cysts identified on preoperative MRI, the arthroscope and instruments should be advanced into the cyst cavity to resect all internal septations, thereby ensuring the entire cyst communicates freely with the joint space.

In this series, all 61 popliteal cysts were successfully treated with arthroscopic internal drainage utilizing the figure-of-four position. Intraoperatively, we found that most knees had one or more intra-articular pathologies, such as meniscal tears (49 cases, 80.32%), cartilage damage (21 cases, 34.42%), and multiple knee pathologies (14 cases, 22.95%). This pattern of associated lesions is consistent with the established literature (Ma et al., 2023; Li et al., 2024), reinforcing the principle that addressing these underlying pathologies is integral to successful cyst management. Our results demonstrate that this technique is both efficient and safe. The mean operative time and mean intraoperative blood loss were comparable to those previously reported for arthroscopic treatment of popliteal cysts (Li et al., 2024; Mei et al., 2023; Sansone & De Ponti, 1999). Most importantly, the procedure yielded excellent functional outcomes. We observed statistically significant improvements in postoperative VAS pain scores, Lysholm knee scores, and R-L grades compared to preoperative baselines, a finding that aligns with outcomes reported by other investigators. As detailed in Table 3, surgical outcomes were particularly favorable for higher-grade cysts. All 21 cases of Grade III popliteal cysts improved after surgery. The Grade II popliteal cysts also decreased from 25 cases preoperatively to 10 cases postoperatively. Asymptomatic recurrence (R-L grade 0-I) was observed in five patients (8.2%), all of whom had presented with high-grade cysts (two Grade II and three Grade III). Notably, these recurrent cysts remained stable in volume and required no further intervention. Consequently, we believed that in these five cases of popliteal cysts, arthroscopic treatment was still effective. It was significantly better than the 17.33% recurrence rate after open surgery for popliteal cysts (de Ruiter et al., 2005).

In summary, arthroscopic internal drainage of popliteal cysts using the figure-of-four position is a technically feasible and safe procedure. Our findings demonstrate significant clinical improvement in pain and function, with a low rate of asymptomatic recurrence at short-to-mid-term follow-up. The figure-of-four position is a valuable technical adjunct that enhances safety and efficiency by improving access and working space in the challenging posteromedial compartment. However, the primary limitation of the study is its retrospective nature, single-arm design, and the absence of a control group, which may have led to potential selection bias. Furthermore, the subjective assessment of the position’s benefits and absence of long-term follow-up data are notable constraints. Therefore, while our results are promising, further research is needed in order to objectively quantify the advantages of the figure-of-four position.

## Supplemental Information

10.7717/peerj.20658/supp-1 Supplemental Information 1 Data.

## Additional Information and Declarations

### Data Availability

The following information was supplied regarding data availability:

The raw data are available in the Supplemental Files.
